# Information Phase Transitions and Epistemic Injustice in Massive Data: Validating the Signal Cliff Based on the Ising Model of Opinion Dynamics

**DOI:** 10.3390/e28060612

**Published:** 2026-05-29

**Authors:** Yasuko Kawahata

**Affiliations:** Faculty of Sociology, Rikkyo University, 3-34-1 Nishi-Ikebukuro, Toshima-ku, Tokyo 171-8501, Japan; ykawahata@rikkyo.ac.jp

**Keywords:** opinion dynamics, Galam model, informational health, epistemic injustice, Signal Cliff, big data, Shannon entropy, phase transition

## Abstract

In the era of big data, the Law of Large Numbers is often treated as an absolute guarantee that increasing sample size (*N*) leads to a more accurate representation of truth. However, this study challenges this paradigm by demonstrating that in social systems characterized by conformity pressure and systemic bias, the maximization of *N* paradoxically triggers a structural shift in the selection and filtration of information. Using a sociophysical framework based on statistical mechanics and opinion dynamics, we identify a critical threshold—the “Signal Cliff”—where the diversity of information plummets and minority signals are irreversibly discarded as statistical noise. By executing large-scale simulations up to $N=10^{10}$ via macro-dynamic approximations, we observe a phase transition from a stochastic phase of informational diversity to a deterministic phase. This collapse of Shannon entropy serves as a mathematical demonstration of “Epistemic Injustice,” where the sheer scale of data acts as a mechanism for silencing minority perspectives. We propose “Informational Health Diagnostics” as a necessary framework for evaluating the integrity of decision-making processes in digital public opinion and democratic elections. This approach provides a vital benchmark for distinguishing between a healthy consensus and a distorted convergence, ensuring robust information judgment in increasingly complex data-driven environments.

## 1. Introduction: Proposing Informational Health Diagnostics and the Epistemological Limits of Massive Data

In contemporary data-driven societies, the Law of Large Numbers stands as a monument of statistical inference, embedding the blind faith that expanding the sample size *N* guarantees improved estimation accuracy and approaches absolute truth. However, in social systems where conformity pressure and social biases are ubiquitous, this premise may structurally collapse. Empirical survey research and big data analyses by Kawahata (2026) suggest that expanding *N* beyond a certain threshold paradoxically erases the faint signals of minorities, driving the system to converge upon a “False Stability”—an informational malfunction [1].

The central question in this paper revolves around “Epistemic Injustice,” a concept originally proposed by Fricker (2007) as testimonial and hermeneutical injustice [2]. This study extends this philosophical concept into the context of computational social science, redefining it as “the mathematical and structural process by which the signals of minorities are irreversibly discarded as mere statistical noise due to data collection algorithms and the tyranny of the Law of Large Numbers”.

To interpret this phenomenon, the application of statistical mechanics to social dynamics provides a robust theoretical foundation. The modeling of consensus and social power has a rich multidisciplinary history, extending from early mathematical sociology models of attitude distribution [3,4,5,6] to comprehensive sociophysics frameworks reviewed by Castellano et al. [7]. Within this extensive domain, the specific frameworks of opinion dynamics pioneered by Galam (1982) based on the Ising model [8] describe the phase transitions of a population through the conflict between interactions (conformity pressure), external magnetic fields (systemic bias), and social temperature (noise) within individual decision-making processes. However, prior sociophysics research has primarily focused on the initial minority proportion $p_{c}$ or consensus-building rules within small groups; the impact of the total population size *N* itself on “information quality” and epistemic injustice has not been sufficiently discussed as a finite-size scaling problem.

The objective of this study is to extend Galam’s initial model to a massive scale, ranging from $N=10^{1}$ to $10^{10}$, and to identify the threshold of informational malfunction—termed the “Signal Cliff”—through extensive computational experiments. The primary parameters used in this simulation and their sociophysical definitions are shown in Table 1.

In this paper, we first empirically demonstrate the collapse of the variance of magnetization (average opinion) accompanying the expansion of *N* using Monte Carlo methods. Subsequently, we analyze the transition of Shannon entropy H(m) using an information-theoretic approach, quantitatively revealing how diversity is structurally rejected in massive data environments. This is not merely the disappearance of statistical error; it serves as mathematical proof of a process where massive *N* sacrifices “Accuracy” and “Diversity” in its pursuit of maximizing “Precision”.

## 2. Review of Previous Research: From Psychometric Biases to Sociophysical Phase Transitions

The theoretical framework of this study is situated at the intersection of micro-cognitive statistics and macro-sociophysics. First, regarding the validity of measurements in Likert scales, Kawahata (2026) proposed a micro-macro linkage model utilizing the Analytic Hierarchy Process (AHP) and the Softmax function, demonstrating that the subjective bias of assessors significantly undermines macro-statistical robustness [9]. This finding suggests that the conventional data science assumption—that “bias cancels out as *N* expands”—does not necessarily hold in non-linear social systems. In the realm of statistical mechanics, the modeling of social dynamics has evolved significantly. Following early consensus formulations [3,5], the introduction of bounded confidence models (e.g., Deffuant et al., Hegselmann and Krause) [10,11] and the Sznajd model’s principle of “united we stand, divided we fall” [12] expanded the understanding of continuous opinion dynamics.

Meanwhile, Galam’s series of studies highlights the structural vulnerabilities of decision-making in big data societies. Galam (2025a) elucidated the process by which hierarchical majority rules irreversibly destroy the fine structure of data, leading to flawed insight extraction [13]. In particular, the discussion of “spontaneous symmetry breaking,” where a minute external magnetic field determines the polarization of the entire system, is crucial for explaining information displacement in platforms with massive *N* [14].

Furthermore, contrarian dynamics, detailed in Galam (2026) and Galam (2025b), corroborate the existence of thresholds where minority opinions are mathematically annihilated [15,16]. Building on these prior studies, this paper integrally analyzes how micro-inputs, defined as “assessor bias,” lead to the “death of information” in large-scale systems through “Ising-like interactions,” approaching the issue from the perspective of informational health diagnostics.

## 3. Transformation of Data Interpretation and Psychometric Significance Through the Identification of the “Signal Cliff”

In standard statistical mechanics, the variance $\sigma^{2}$ of a macroscopic observable in a mean-field system scales strictly as $\sigma^{2}\propto 1/N$, provided the system is not exactly at a critical point. Our model intrinsically obeys this fundamental scaling law. The “Signal Cliff” identified in this study is the profound sociopolitical consequence of this very scaling as a finite-size effect. In a stochastic system not at a critical point, finite-size fluctuations allow the system to temporarily explore minority states. However, when conformity pressure *J* and a symmetry-breaking systemic bias *H* intervene, the exponential decay of these finite-size fluctuations (driven by 1/N) irreversibly traps the system into the deterministic macroscopic attractor. Consequently, the information held by minority opinions disappears discontinuously once a critical population size $N_{c}$ is crossed.

Because parameters such as conformity pressure *J* and systemic bias *H* are impossible to observe directly in real-world social survey data (e.g., 5-point Likert scales), overcoming this limitation requires structuring the model as an “Inverse Problem” within the implementation framework of informational health diagnostics. Specifically, upon detecting abnormal variance shrinkage or entropy collapse in acquired real data, combining this simulation model with Bayesian inference allows for the back-calculation (estimation) of the hidden intensities of *J* and *H*.

The advantages of identifying this “Signal Cliff” for data interpretation are summarized in the Table 2.

## 4. Overcoming Computational Costs in Opinion Dynamics and Establishing Benchmarks

In the research domain of opinion dynamics, identifying behavior when the system size *N* is maximized has been extremely difficult due to computational complexity. Particularly in conventional Monte Carlo methods that sequentially calculate individual spin flips, computations on a scale of $N=10^{10}$ cannot be completed within realistic timeframes. This study achieves a significant reduction in computational cost by employing the following mathematical techniques, rooted in Galam’s (1982) [8,10,11,12].

- Introduction of Mean-Field Approximation: Aggregates interactions with all agents into a mean field *m*, reducing computational complexity from $O(N^{2})$ to O(N).
- Transition to Macro-Dynamics: In regions where *N* is massive, the binomial distribution B(N,p) is approximated by the normal distribution N(Np,Np(1−p)), constructing a continuous-value simulation that bypasses individual agent updates.

The thresholds identified through these techniques serve as benchmarks for “computational cost preparation” and “prediction accuracy” when scaling other opinion dynamics models (such as the q-voter or Sznajd models) for large-scale societal implementation.

## 5. The “Signal Cliff” in Digital Public Opinion and Elections: Potential Application as an Anomaly Threshold

Recently, the impact of information diffusion on social media upon election results and macro public opinion formation has reached an undeniable scale. In particular, when specific political biases or conformity pressures are amplified in digital spaces, there is a pointed risk that the obtained outcome is not an “aggregation of public will” but a “structural displacement” caused by informational malfunction. The “Signal Cliff” identified in this study serves as a quantitative threshold to determine whether the observed outcomes in such large-scale decision-making processes constitute statistical anomalies (Table 3).

Decision-making by majority rule leads to the correct conclusion via the Law of Large Numbers only if the independence of individual judgments is guaranteed (Condorcet’s Jury Theorem). However, as suggested by the Galam model, in systems where strong conformity pressure *J* exists, a massive sample size *N* accelerates the “death of information”.

## 6. Mathematical Structure of Large-Scale Opinion Dynamics Simulations

This paper explicates the theoretical background of the simulation code that mathematically and physically verifies the malfunction of “Epistemic Injustice” and “Informational Health” in data science. Modern big data faith holds the implicit assumption that expanding the sample size *N* asymptotically approaches the true distribution. However, this computational experiment clarifies that in social spaces with conformity pressure and minor systemic biases, the maximization of *N* paradoxically creates a threshold (Signal Cliff) that structurally conceals minority signals and induces False Stability (see Appendix A).

### 6.1. Model Formulation and Large-Scale Computational Approximations

This simulation is formulated as a Monte Carlo simulation based on Glauber dynamics utilizing a mean-field approximation. Each agent possesses a spin $S_{i}\in \left\{+1,-1\right\}$, and *m* represents the magnetization (average opinion, a continuous value in [−1,1]) of the entire system. At a given time step, the probability $p_{+}$ that an arbitrary agent selects the majority opinion (+1) follows a statistical mechanical Boltzmann distribution:

(1) $$p_{+}=\frac{1}{1+\exp\left(-2\beta (Jm+H)\right)}$$

The dynamic approximation switching enables simulations at a global scale of $N=10^{10}$. At small to medium scales where $N<10^{6}$, exact binomial sampling is performed:

(2) $$n_{+}∼B\left(N,p_{+}\right)$$

At massive scales where $N\geq 10^{6}$, the Central Limit Theorem is applied:

(3) $$\mu =Np_{+},\;\sigma =\sqrt{Np_{+}\left(1-p_{+}\right)}$$

To ensure this study is fully self-contained and reproducible without relying solely on external repositories, we explicitly state the simulation parameters. The experiments evaluated the system using a mean-field topology to establish a theoretical baseline. We fixed the parameters to simulate a society slightly removed from the critical temperature, incorporating a marginal systemic bias: rescaled temperature βJ=1.0 and external magnetic field H=0.01. Under these fixed conditions, we swept the population size *N* logarithmically from $10^{1}$ to $10^{10}$ to observe the variance collapse.

The onset point of this Signal Cliff is deduced to follow an exponential scaling law, such as $N_{c}\propto \exp\left(C\cdot J/T\right)$.

### 6.2. Loss of Stochastic Fluctuations and Deterministic Synchronization

In this paper, we discuss the simulation results based on the mathematical validation of “Epistemic Injustice” proposed by Kawahata (2026) [17]. The statistical test results vividly demonstrate the process of “Statistical Tyranny,” transforming from a micro “Stochastic System” to a macro “Deterministic System”.

**Figure 1:**
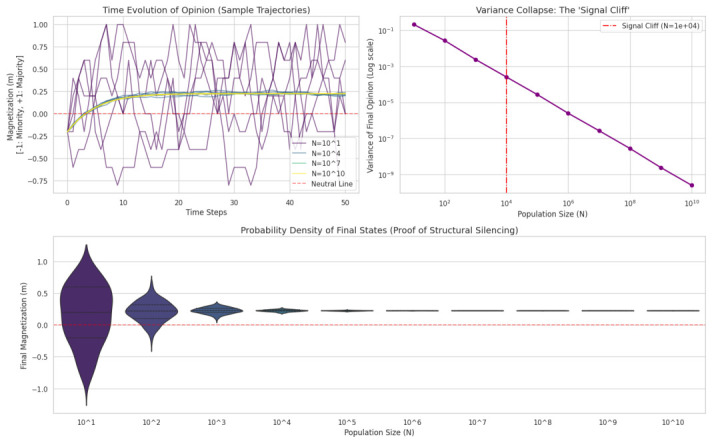
Opinion Transition and Variance Collapse with Expanding System Size *N*.

The occurrence of the “Signal Cliff” at $N=10^{4}$, shown in Figure 1, sounds an extremely important alarm for the practice of social surveying. Maximizing *N* within the framework of the Galam model is a process that exploits the Law of Large Numbers to mathematically reject minority opinions.

### 6.3. Phase Transitions and Informational Death

**Figure 2:**
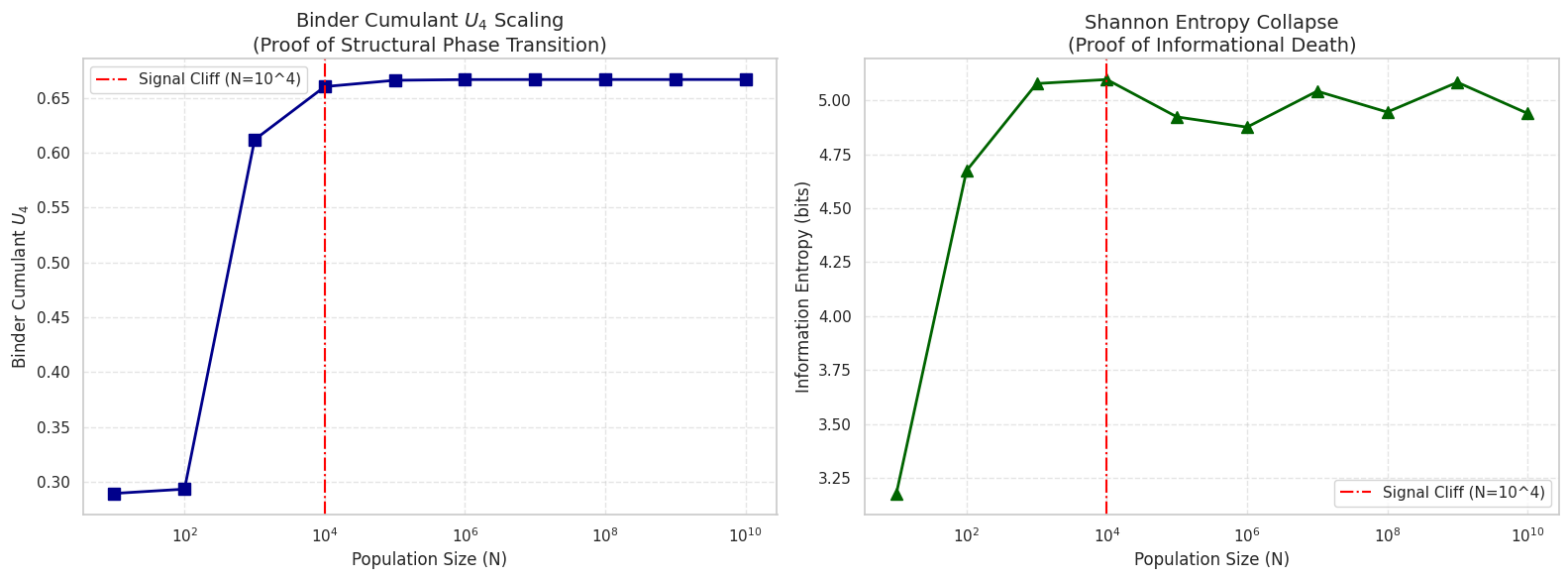
Scaling of the Binder Cumulant $U^{4}$ and Collapse of Shannon Entropy.

The structural phase transition and “Informational Death” brought about by the expansion of system size *N* is clearly illustrated in the Figure 2.

The Binder Cumulant ($U_{4}$)’s sharp rise crossing $N=10^{4}$ means the system has undergone an irreversible structural transition to an order-dominated phase [18].

## 7. Discussion: Topology and Characteristic Scales

While this study utilized a mean-field approximation for computational feasibility and theoretical baselining, the characteristic scale $N_{c}$ at which the Signal Cliff occurs is highly dependent on the underlying network topology. As established in the statistical physics of social dynamics [7], the scaling behavior and phase transitions vary significantly if the system is placed on a regular lattice, a scale-free network, or a small-world topology. In networks with high local clustering, minority clusters may survive longer against the mean-field bias. Future research must couple these topological dependencies with inverse-problem analyses on real-world survey data to map the exact boundaries of epistemic injustice in diverse social structures.

## 8. Conclusions

The structural changes occurring at $N=10^{4}$ transcend the framework of sociophysics, providing profound critical grounding against data-driven societies [19,20,21].

## Figures and Tables

**Table 1:** Parameters and Sociophysical/Informational Definitions.

Parameter	Sociophysical and Informational Definition
*N*	System size (total population). Determines the resolution and statistical precision.
*J*	Conformity pressure (coupling constant). The strength of interaction aiming for opinion alignment.
*H*	External magnetic field. A systemic bias leaning toward the majority.
β	Inverse social temperature (1/T). Represents decision confidence or inverse noise level.
H(m)	Shannon entropy. An indicator of informational diversity and health.

**Table 2:** Utility of the Signal Cliff in Data Interpretation.

Item	Utility in Data Interpretation
Distinguishing False Stability	Verifies whether the high “Precision” obtained by massive *N* is actually “Distorted Accuracy” caused by social pressure, allowing for the estimation of hidden bias *H*.
Identifying Diversity Thresholds	Knowing the boundary $N_{c}$ where minority warning signals are mathematically erased enables the design of appropriate survey scales.
Visualizing Injustice	Objectively proves the process by which opinions with specific attributes are structurally rejected via entropy collapse.

**Table 3:** Function of the Signal Cliff as an Anomaly Threshold.

Detection Target	Function as an Anomaly Threshold
Identifying False Enthusiasm	When magnetization *m* rapidly reaches consensus, if the collapse rate of entropy exceeds the theoretical cliff, it suggests manipulation by algorithms or peer pressure.
Discontinuous Phase Transitions	In election results, if convergence significantly falls below the variance expected at the specific *N* scale, it can be detected as an anomalous point.
Evaluating Resolution of Public Will	Assesses whether digital bias acted as *H* to mathematically silence genuine minority opinions, reverse-calculating this from the residual Shannon entropy.

## Data Availability

The Python code and simulation frameworks supporting the findings of this study are openly available on GitHub at the following repository: https://github.com/RUDATAScience/epistemicinjustice-validation (accessed on 26 April 2026).

## Abbreviations

    The following abbreviations are used in this manuscript:

AHP	Analytic Hierarchy Process
KL	Kullback–Leibler
NLP	Natural Language Processing
ABM	Agent-Based Model
CLT	Central Limit Theorem

## Appendix A. Computational Environment and High-Speed Reproducibility

To ensure mathematical reproducibility, the specific computational environment and execution metrics are detailed. The simulations were executed on a standard cloud computing instance.

**Table A1:** Hardware Specifications for Large-Scale Simulation.

Item	Specification
Architecture	x86_64 (Little Endian)
CPU	Intel(R) Xeon(R) CPU @ 2.20 GHz
Core Count	2 vCPUs
L3 Cache	55 MiB
OS	Linux (64-bit support)

### Algorithmic Optimization and Execution Metrics

The simulation was implemented in Python 3.10. A defining feature is the mathematical restructuring of the opinion dynamics process.

- Computational Complexity and Macro-Approximation: Conventional ABMs require O(N) looping structures. To overcome this, our framework transitions to a macro-dynamic approach for $N\geq 10^{6}$.
- Efficiency Metrics: This architectural optimization reduces the time complexity to O(1). Consequently, the core simulation for any given population completes in approximately 0.5 s.
- Reproducibility: The pseudo-random number generator seed was explicitly fixed (e.g., numpy.random.seed(42)).
